# Long-Term Outcomes of Patients With Cocaine Use Disorder: A 18-years Addiction Cohort Study

**DOI:** 10.3389/fphar.2021.625610

**Published:** 2021-02-18

**Authors:** Arantza Sanvisens, Anna Hernández-Rubio, Paola Zuluaga, Daniel Fuster, Esther Papaseit, Sara Galan, Magí Farré, Robert Muga

**Affiliations:** ^1^ Department of Internal Medicine, Hospital Universitari Germans Trias i Pujol-IGTP, Universitat Autónoma de Barcelona, Department of Medicine, Badalona, Spain; ^2^ Department of Clinical Pharmacology, Hospital Universitari Germans Trias i Pujol-IGTP, Universitat Autònoma de Barcelona, Badalona, Spain

**Keywords:** cocaine use disorder, VACS index, comorbidity, mortality, hospitalization

## Abstract

**Objective:** Cocaine Use Disorder (CUD) has been associated with multiple complications and premature death. The purpose of the present study was to analyze the relationship between baseline medical comorbidity and long-term medical outcomes (i.e., hospitalization, death) in a cohort of patients primarily admitted for detoxification. In addition, we aimed to analyze cause-specific mortality.

**Methods:** longitudinal study in CUD patients admitted for detoxification between 2001 and 2018. Substance use characteristics, laboratory parameters and medical comorbidity by VACS Index were assessed at admission. Follow-up and health-related outcomes were ascertained through visits and e-health records. Kaplan-Meier and Cox regression models were used to analyze survival and predictors of hospitalization and death.

**Results:** 175 patients (77.7% men) were included. Age at admission was 35 years [IQR: 30–41 years], 59.4% of the patients being intranasal users, 33.5% injectors, and 7.1% smokers. Almost 23% of patients had concomitant alcohol use disorder, 39% were cannabis users and 9% opiate users. The median VACS Index score on admission was 10 points [IQR: 0–22]. After 12 years [IQR: 8.6–15 years] of follow-up there were 1,292 (80.7%) ED admissions and 308 (19.3%) hospitalizations. The incidence rate of ED admission and hospitalization was 18.6 × 100 p-y (95% CI: 15.8–21.8 × 100 p-y). Mortality rate was 1.4 × 100 p-y (95% CI: 0.9–2.0 × 100 p-y) and, baseline comorbidity predicted hospitalization and mortality: those with VACS Index >40 were 3.5 times (HR:3.52, 95% CI: 1.19–10.4) more likely to dye with respect to patients with VACS < 20.

**Conclusion:** addiction care warrants optimal stratification of medical comorbidity to improve health outcomes and survival of CUD patients seeking treatment of the disorder.

## Introduction

Cocaine is the second most widely used illegal drug in Western Europe after cannabis. According to the European Monitoring Center for Drugs and Drug Addiction (EMCDDA), about four million people aged 15–64 have used cocaine in the last year, and the number of users has increased in recent years (European Monitoring Center for Drugs and Drug Addiction (EMCDDA), 2019).

According to the EDADES population survey on alcohol and drugs, 2% of the general population aged 15–64 in Spain uses cocaine (Observatorio Español de las Drogas y las Adicciones, 2019). Moreover, 54% of people who have used cocaine in the last year have used it in the last month (Observatorio Español de las Drogas y las Adicciones, 2019). In Catalonia (Spain), 24% of people who seek treatment for substance use disorder (SUD) have a cocaine use disorder (CUD), and this percentage has increased in recent years (Subdirecció General de Drogodependències, 2018).

CUD has been associated with serious systemic complications, frequent use of healthcare resources (i.e., emergency department (ED) admissions, hospitalization), and premature death (Degenhardt et al., 2011; Butler et al., 2017). In fact, cocaine use can aggravate inflammatory diseases and alter immune functions that favor the progression of cardiovascular, respiratory, or infectious diseases (Taylor et al., 2016; Bachi et al., 2017). In addition, it has been communicated that cocaine intoxication can cause acute kidney injury, hepatotoxicity, and disseminated intravascular coagulation (Vitcheva, 2012; Filho et al., 2019). On the other hand, cocaine use has been associated with an increased risk of HIV infection and hepatitis C virus (HCV) infection even in the absence of injecting drug use (Macías et al., 2008; Deiss et al., 2012).

In addition, patients with CUD show a high prevalence of psychiatric comorbidities, such as mood disorders (12–62%), anxiety disorders (21–45%) and suicidal tendencies, among others (Vergara-Moragues et al., 2012; Warden et al., 2016). Cocaine use has also been associated with traffic accidents and violence (Giovanardi et al., 2005; Pavarin et al., 2011).

Polysubstance use is common among cocaine users, especially alcohol consumption, but the concurrent use of marijuana and opiates is also prevalent, and has been associated with poor health outcomes (Stinson et al., 2005; Timko et al., 2018). In the US, the second wave of the National Epidemiologic Survey on Alcohol and Related Conditions (NESARC) estimated that 79% of people with CUD have a concomitant alcohol use disorder (AUD) (Stinson et al., 2005).

A recent systematic review and meta-analysis on healthcare utilization demonstrated that in SUD patients, hospitalization and ED admissions are 5 and 7 times more frequent, respectively, compared to the general population (Lewer et al., 2020). In addition, the death rate of patients with CUD ranges from 0.5 to 6.2 × 100 person-years (p-y) and is considered to be 4 to 8 times higher than the death rate of the general population (Arendt et al., 2011; Degenhardt et al., 2011).

Our hypothesis is that the chronicity of CUD is suggestive of the presence of multiple medical complications, which leads to the excessive use of healthcare resources (i.e., ED visits and hospitalization). We aimed to analyze the relationship between baseline medical comorbidity, use of health resources, and long-term health outcomes among those seeking treatment for CUD.

## Materials and Methods

This was a longitudinal study of patients admitted to the addiction treatment unit of a tertiary hospital (Germans Trias i Pujol University Hospital) between January 2001 and May 2018. The unit admits patients diagnosed with SUD in an area in the north of Barcelona (Spain) with 400,000 inhabitants. There were 837 admissions for addiction treatment between January 2001 and May 2018, of which 195 (23.3%) were due to CUD in 175 patients. In those who were admitted more than once, only the first admission was analyzed.

The patients came from local primary care centers and from two municipal outpatient addiction clinics, one in Badalona (250,000 inhabitants) and the other in Santa Coloma de Gramenet (150,000 inhabitants), both located in the metropolitan area of Barcelona (Spain). The main admission criteria for CUD treatment were as follows: failure in outpatient treatment, concurrent dependency on other addictive drugs or alcohol, serious concurrent medical problems, severe impairment of psychosocial functioning, lack of family and social support, and use of crack or freebase cocaine or intravenous cocaine abuse, among others.

All patients received a diagnosis of CUD according to the Diagnostic and Statistical Manual of Mental Disorders, fourth Ed (DSM-IV) and fifth Ed (DSM-5). Due to the transition from DSM-IV to DSM-5, not all participants were evaluated under the same DSM.

On admission, data on the use of cocaine and other substances (i.e., alcohol, cannabis, opiates) were collected, including age of onset, route of administration, and duration. DSM criteria were used to diagnose AUD. Cannabis and opiates use was ascertained through urinalysis at admission. For the purposes of this study, patients were classified according to the route of cocaine administration as either intranasal or non-intranasal users (i.e., injectors, smokers).

In all patients, blood samples were drawn for biochemical and hematological parameters, and for serologic testing for human immunodeficiency virus (HIV) infection and hepatitis C virus (HCV) infection. Anthropometric data (i.e., height and weight) were obtained as well.

Medical comorbidity on admission was analyzed using the VACS Index (Veterans Aging Cohorts Study Index). VACS Index assigns a score based on age and on blood parameters, such as hemoglobin, platelets, aspartate and alanine aminotransferase levels, creatinine, HIV infection, CD4 lymphocytes, HIV RNA, and HCV infection. The VACS Index ranges from 0 to 164 points, with a higher score indicating greater comorbidity. The VACS Index has been associated with an increased risk of hospitalization and death in patients with and without HIV infection (Blackstock et al., 2013; Justice et al., 2013; Tate et al., 2013). In HIV-negative patients, HIV RNA is considered undetectable.

### Follow-Up, Comorbidity and Outcomes

The patients were followed up until September 30, 2018 through in-person visits and review of the ED visits and hospitalization e-health records of the Catalan health department. The diagnoses made during the ED visits and hospitalization were coded according to the 10th revision of the International Classification of Diseases (ICD-10). The diagnostic coding was carried out by two members of the research team (AS and RM) independently; coding discrepancies were resolved by consensus.

Mortality was analyzed by cross-referencing the data with the National Death Index as of September 30, 2018. The causes of death were established by reviewing the medical history.

### Ethics

All patients gave written informed consent, and the study design was approved by the Ethics Committee of the Germans Trias i Pujol University Hospital (approval number PI-13-082). The methods were in compliance with the ethical standards for medical research and the principles of good clinical practice in accordance with the World Medical Association’s Declaration of Helsinki.

### Statistical Analysis

Descriptive statistics were expressed as median and interquartile range (IQR) for quantitative variables and as absolute frequencies and percentages for qualitative variables.

We used the chi-square test to detect significant differences in qualitative variables and t-Student test for differences in quantitative variables. The Kruskal-Wallis equality-of-populations rank test and Mann-Whitney U test were used to analyze differences in the distribution of episodes during follow-up.

Rates were calculated in p-y by dividing the number of observed events during the study period by the sum of all individual follow-up times. The survival estimates were analyzed using the Kaplan-Meier curves. Cox regression models were used to analyze the risk factors of first hospitalization after discharge and mortality. The sex, variables related to substance use, and the VACS Index score were included in the analysis. All covariates that were statistically significant in the univariate analysis were included in the multivariate analysis. Prior to implementing the statistical models, we checked the proportional hazard assumptions using tests and graphs based on the Schoenfeld residuals.

*p*-values <0.05 were considered statistically significant. Statistical analysis was performed using Stata software (version 11.1, College Station, Texas, United States).

## Results

### Patient Characteristics at Baseline

The study included 175 patients (77.7% men) aged 35 years [IQR: 30–41 years]. The age of onset of cocaine use was 22 years [IQR: 18–26 years], with 59.4% of the patients being intranasal users, 33.5% injectors, and 7.1% smokers. Moreover, 22.9% of the patients had concomitant AUD. According to the screening for illegal drugs in urine samples, 39.4% were cannabis users and 9% opiate users. Overall, 58.1% of the patients used alcohol or other substances in addition to cocaine. The most common combinations of substances were cocaine and cannabis (27.5%), cocaine and alcohol (14.4%), and cocaine, alcohol, and cannabis (7.5%).

The laboratory test results for hemoglobin, total cholesterol, and gamma-glutamyl transferase were 14.2 g/dl [IQR: 12.9–15.3 g/dl], 170 mg/dl [IQR: 147–201 mg/dl], and 28 U/L [IQR: 17–62 U/L], respectively. Moreover, 24.7% of the patients were HIV-positive and 47.4% were anti-HCV positive.

The body mass index (BMI) was 19.5 kg/m^2^ [IQR: 16.4–23.0 kg/m^2^]. The median VACS Index score on admission was 10 points [IQR: 0–22; range 0–69]. Table 1 shows the sociodemographic characteristics, alcohol and substance use, and clinical and blood parameters.

**TABLE 1:** Sociodemographic characteristics, alcohol and substance use, and clinical and blood parameters in 175 patients admitted for the treatment of CUD in metropolitan Barcelona, Spain.

	N = 175
Men, *n* (%)	136 (77.7)
Age, median [IQR]	35 [30–41]
BMI, (kg/m^2^) (*n* = 144), median [IQR]	19.5 [16.4–23.0]
Drug related parameters	*n* (%)
Age at starting cocaine use, median [IQR]	22 [18–26]
Route of cocaine administration (*n* = 170)	
Injected	57 (33.5)
Intranasal	101 (59.4)
Smoked	12 (7.1)
Duration of CUD (months) (*n* = 164), median [IQR]	111 [36–180]
AUD	40 (22.9)
Urine screening at admission	
Opiates (*n* = 158)	14 (8.9)
Cannabis (*n* = 160)	63 (39.4)
Antecedent of injection drug use (*n* = 172)	82 (47.7)
Hematology parameters	Median [IQR]
Platelet count (x10^9^L) (*n* = 174)	219 [184–270]
Hemoglobin (g/dL) (*n* = 174)	14.2 [12.9–15.3]
Biochemistry	Median [IQR]
Creatinine (mg (dL) (*n* = 173)	0.9 [0.78–0.99]
Glomerular fíltration rate (*n* = 173)	97.6 [83.9–109]
Aspartate aminotransferase (U/L)	24 [18–40]
Alanine aminotransferase (U/L) (*n* = 173)	25 [16–46]
Comorbidity	n (%)
HIV Infection (*n* = 174)	43 (24.7)
CD4^+^ T cell (x10^9^L), median [IQR]	457 [214–730]
Viral load (copies/mL), median [IQR]	1,000 [49–22,000]
HCV Infection (*n* = 173)	82 (47.4)
FIB-4 (*n* = 173), median [IQR]	0.76 [0.54–1.28]
Advanced liver fibrosis (>3.25)	8 (4.6)
VACS index (*n* = 173), median [IQR]	10 [0–22]
<20	123 (71.1)
20–39	38 (22.0)
>40	12 (6.9)

AUD, alcohol use disorder; BMI, body mass index; CUD, cocaine use disorder; HCV, hepatitis C virus; HIV, human immunodeficiency virus; IQR, interquartile range.

### Follow-Up, ED Visits, Hospitalizations and Comorbidity

Table 2 shows the socio-demographics, substance use characteristics, and clinical and blood parameters of CUD patients that were hospitalized during follow-up. Hospitalization was significantly more frequent in women (*p* = 0.049), in those with antecedent of injecting drug use (*p* = 0.028), and in HIV-positive and HCV-positive patients (*p* = 0.001 and *p* = 0.047, respectively).

**TABLE 2:** Baseline characteristics of CUD patients according to hospitalization and death during follow-up.

	Hospitalization			Death		
	Yes	No	*p* value	Yes	No	*p* value
	N = 97	N = 78		N = 27	N = 148	
Men, *n* (%)	70 (72.2)	66 (84.6)	0.049	21 (77.8)	115 (77.7)	0.993
Age, median [IQR]	36 [31–41]	34.5 [30–40]	0.392	37 [32–42]	34.5 [30–40.5]	0.323
BMI, (kg/m^2^) (*n* = 144), median [IQR]	18.5 [15.6–22.0]	21.0 [17.6–23.4]	0.030	19.4 [14.6–22.7]	19.6 [16.7–23.1]	0.334
Drug related parameters						
Age at starting cocaine use, median [IQR]	21 [17–26]	19.5 [18–25]	0.484	22 [18–24]	20 [17–26]	0.963
Route of cocaine administration (*n* = 170)						
Injected	33 (35.5)	24 (31.2)	0.509	17 (63.0)	40 (28.0)	0.001
Intranasal	52 (55.9)	49 (63.6)		10 (37.0)	91 (63.6)	
Smoked	8 (8.6)	4 (5.2)		0 (0)	12 (8.4)	
Duration of CUD (months) (*n* = 164), median [IQR]	120 [42–180]	96 [36–180]	0.968	96 [36–204]	114 [48–180]	0.604
AUD	25 (25.8)	15 (19.2)	0.306	4 (14.8)	36 (24.3)	0.279
Urine screening at admission						
Opiates (*n* = 158)	8 (9.1)	6 (8.6)	0.909	3 (13.0)	11 (8.1)	0.445
Cannabis (*n* = 160)	34 (37.8)	29 (41.4)	0.639	12 (50.0)	51 (37.5)	0.248
Antecedent of injection drug use (*n* = 172)	52 (55.3)	30 (38.5)	0.028	21 (80.8)	61 (41.8)	<0.001
*H*ematology parameters						
Platelet count (×10^9^L) (*n* = 174)	226 [189–279]	210 [181–262]	0.148	193 [139–233]	224 [191–272]	0.005
Hemoglobin (g/dL) (*n* = 174)	14.2 [13–15.2]	14.1 [12.9–15.3]	0.861	14.7 [12.6–15.1]	14.1 [13.0–15.3]	0.973
Biochemistry						
Creatinine (mg (dL) (*n* = 173)	0.89 [0.78–0.99]	0.9 [0.78–0.97]	0.876	0.9 [0.74–1.01]	0.89 [0.79–0.99]	0.955
Glomerular fíltrate rate (*n* = 173)	96.5 [82.8–107.5]	98.3 [85.7–112.4]	0.248	97.7 [83.7–112.4]	97.6 [84.1–108.4]	0.997
Aspartate aminotransferase (U/L)	25 [19–39]	23 [17–41]	0.429	38 [22–73]	23 [18–36.5]	0.006
Alanine aminotransferase (U/L) (*n* = 173)	25.5 [16–43.5]	22 [15–51]	0.877	42 [21–65]	23 [15–42]	0.109
Comorbidity						
HIV Infection (*n* = 174)	33 (34.4)	10 (12.8)	0.001	14 (51.8)	29 (19.7)	<0.001
CD4^+^ T cell (x10^9^L), median [IQR]	486 [242–771]	382 [202–562]	0.314	364 [202–457]	562 [242–792]	0.097
Viral load (copies/mL), median [IQR]	2,700 [49–22,000]	337 [49–8,100]	0.325	4,050 [49–15,000]	570 [49–22,000]	0.725
HCV Infection (*n* = 173)	52 (54.2)	30 (39.0)	0.047	21 (77.8)	61 (41.8)	0.001
FIB-4, median [IQR] (*n* = 173)	0.76 [0.54–1.30]	0.78 [0.53–1.26]	0.732	1.5 [0.8–2.2]	0.7 [0.5–1.1]	<0.001
Advanced liver fibrosis (>3.25)	3 (3.1)	5 (6.5)	0.294	4 (14.8)	4 (2.7)	0.006
VACS index, median [IQR] (*n* = 173)	10 [0–27]	10 [0–15]	0.110	21 [11–33]	10 [0–19]	0.001
<20	64 (66.7)	59 (76.6)	0.349	13 (48.1)	110 (75.3)	0.005
20–39	24 (25.0)	14 (18.2)		9 (33.3)	29 (19.9)	
>40	8 (8.3)	4 (5.2)		5 (18.5)	7 (4.8)	

AUD, alcohol use disorder; BMI, body mass index; CUD, cocaine use disorder; HCV, hepatitis C virus; HIV, human immunodeficiency virus; IQR, interquartile range.

**FIGURE 1:**
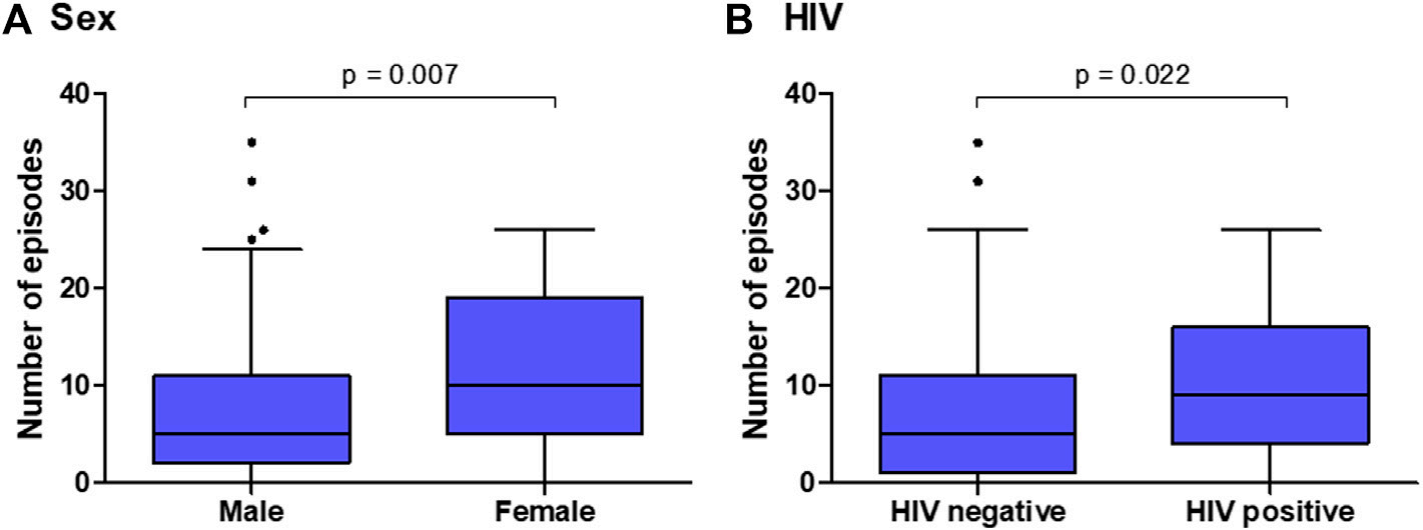
ED admissions or hospitalizations by **(A)** sex and **(B)** HIV infection status in a cohort of 175 patients admitted for treatment of CUD in metropolitan Barcelona, Spain.

**FIGURE 2:**
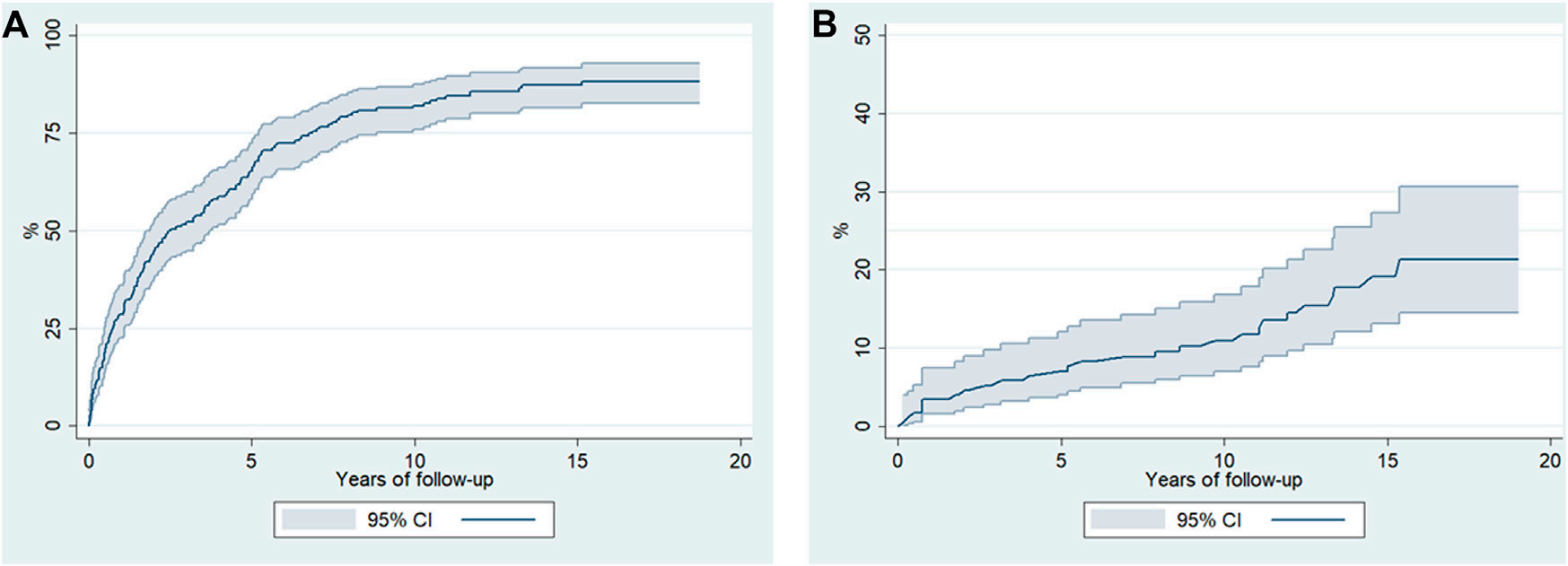
Kaplan-Meier estimates (95% confidence intervals) for **(A)** ED visits or hospitalization episodes of 175 CUD patients and for **(B)** survival after being admitted for detoxification in metropolitan Barcelona, Spain.

The median follow-up time was 12.1 years [IQR: 8.6–15.1 years] with a total time of 1,973.2 p-y. At the end of the study, there were 1,292 (80.7%) ED admissions and 308 (19.3%) hospitalizations. The median number of ED admissions and hospitalizations per patient were 5 [IQR: 1–10] and 1 [IQR: 0–2], respectively. The vast majority (85.1%) of the patients presented at least one episode of ED admission or hospitalization. Statistically significant differences were observed in the distribution of episodes (either ED admission or hospitalization) according to gender and HIV status; specifically, women (z = −2.704, *p* = 0.007) and HIV-positive patients (z = −2.291, *p* = 0.022) had a greater number of episodes (Figure 1). The probability of having an ED admission or hospitalization was 50% after 2.5 years (95% CI: 1.9–3.7 years) (Figure 2A).

**FIGURE 3:**
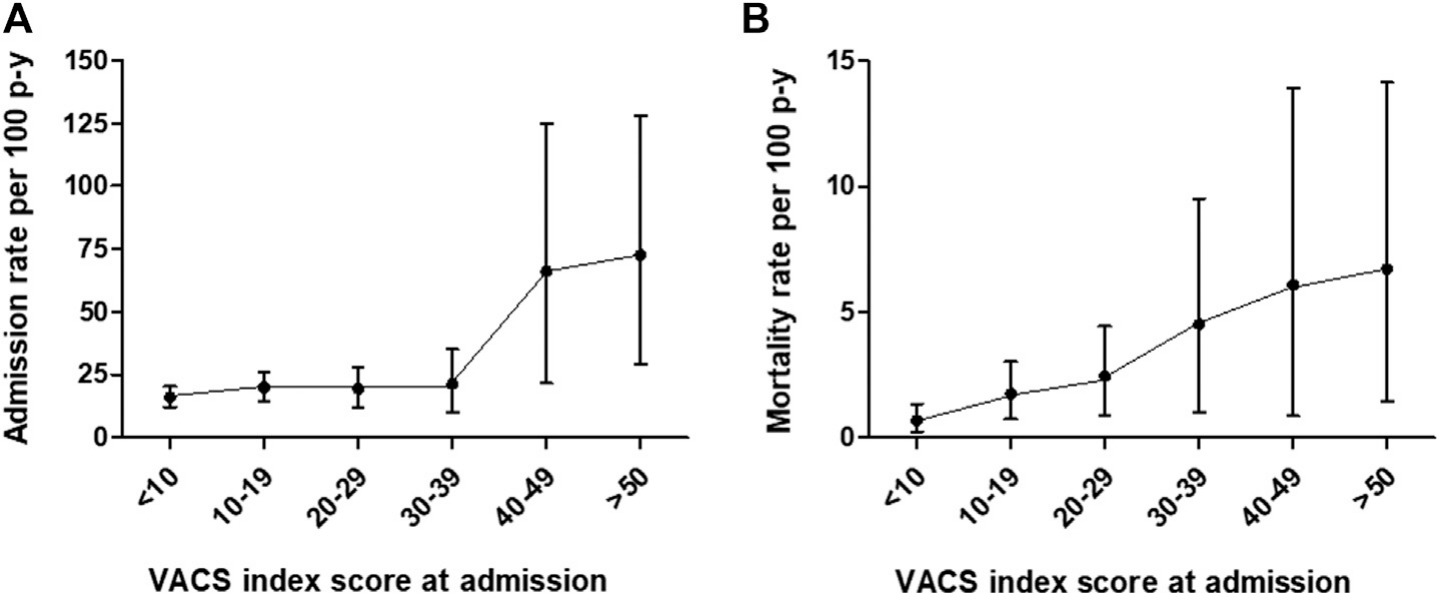
Comorbidity (VACS Index)-adjusted incidence rates (95% confidence intervals) of **(A)** ED admission or hospitalization episodes and **(B)** mortality in a cohort of 175 patients seeking treatment of CUD in metropolitan Barcelona, Spain.

The incidence rate of ED admission or hospitalization was 18.6 × 100 p-y (95% CI: 15.8–21.8 × 100 p-y), which was significantly higher in women (rate ratio (RR): 1.8, 95% CI: 1.22–2.60, *p* = 0.002) and in patients with concomitant AUD (RR: 2.0, 95% CI: 1.3–2.9, *p* < 0.001). Figure 3A shows the incidence of episodes according to baseline medical comorbidity.

In terms of ED admissions, 19% were related to trauma/injuries (i.e., fractures, contusions, wounds), 19% to non-specific/unclassified symptoms, 10% to substance use, and 8.5% to mental disorder.

**FIGURE 4:**
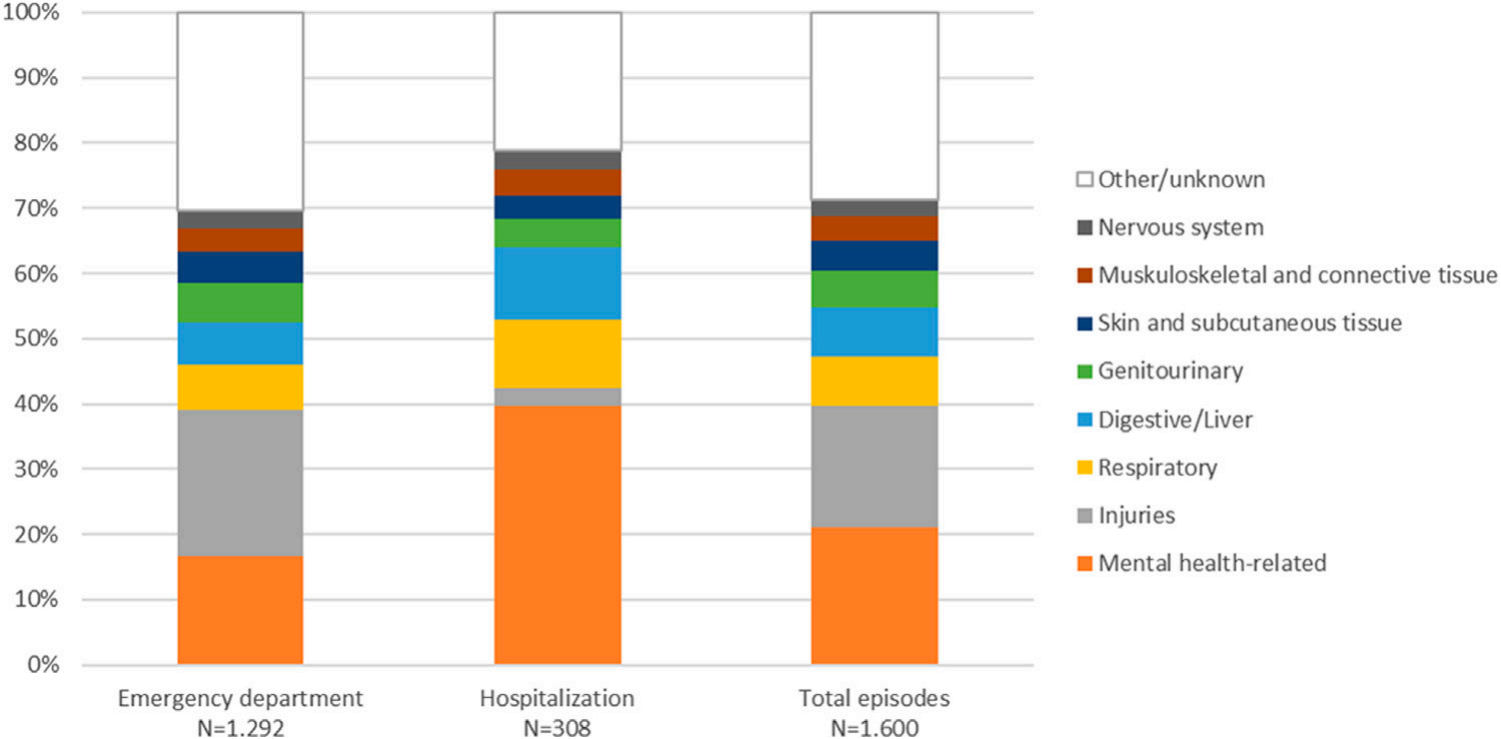
Distribution of 1,292 ED admissions and 308 hospitalizations according to ICD-10 diagnostic codes.

Regarding hospitalization, almost 40% of the episodes were related to mental health, 11% to the liver/digestive system, and 10.4% to respiratory conditions (i.e., pneumonia). Figure 4 shows the distribution of ED admission and hospitalization episodes according to the main diagnosis.

Table 3 shows the risk factors for hospitalization. Specifically, being women (hazard ratio (HR): 1.61, 95% CI: 1.02–2.58), presenting concomitant AUD (HR: 1.42, 95% CI: 1.18–1.71), and having VACS Index >40 (HR: 2.59, 95% CI: 1.13–5.94) were significantly associated with a higher probability of hospitalization.

**TABLE 3:** Cox regression model for predictors of hospitalization and death in a cohort of 175 patients admitted for treatment of CUD in metropolitan Barcelona, Spain.

	Hospitalitzation		Death	
	Unadjusted HR (95% CI)	Multivariate HR (95% CI)	Unadjusted HR (95% CI)	Multivariate HR (95% CI)
Women	**1.89 (1.21–2.96)**	**1.61 (1.01–2.58)**	1.07 (0.43–2.65)	
Age at starting cocaine use: 5 years increase	1.15 (0.93–1.43)		0.93 (0.57–1.52)	
Non-intranasal cocaine	0.94 (0.62–1.41)		**2.30 (1.05–5.03)**	1.64 (0.69–3.92)
Duration of CUD	0.99 (0.99–1.00)		1.00 (0.99–1.01)	
AUD	**1.23 (1.05–1.44)**	**1.27 (1.08–1.48)**	0.96 (0.67–1.38)	
Urine screening at admission				
Opiates	0.72 (0.35–1.50)		1.28 (0.38–4.32)	
Cannabis	0.84 (0.55–1.29)		1.49 (0.67–3.32)	
VACS index				
<20	1	1	1	1
20–39	1.30 (0.81–2.08)	1.24 (0.77–2.02)	**2.39 (1.02–5.59)**	1.86 (0.73–4.72)
>40	**2.63 (1.25–5.52)**	**2.64 (1.20–5.83)**	**4.35 (1.55–12.2)**	**3.52 (1.19–10.4)**

Statistically significant differences are in bold. AUD, alcohol use disorder; CUD, cocaine use disorder; CI, confidence interval; HR: hazard ratio.

### Mortality and Causes of Death

Of the patients included in the study, 15.4% (*n* = 27) died during the follow-up and the death rate was 1.4 × 100 p-y (95% CI: 0.9–2.0 × 100 p-y). Table 2 shows the differences between those who died and those who survived; all-cause mortality was significantly associated with current or past injecting drug use (*p* = 0.001 and *p* < 0.001, respectively), HIV infection (*p* < 0.001), HCV infection (*p* < 0.001), advanced liver fibrosis (*p* = 0.006) and higher scores in VACS Index (*p* = 0.005).

Figure 2B shows the estimate (Kaplan-Meier) of survival after admission for CUD treatment. The multivariate analysis showed that medical comorbidity was the only predictor of death; patients with VACS >40 showed 3.5 times greater probability of death (HR 3.52, 95% CI: 1.19–10.4) compared to patients with VACS Index <20 (Table 3). Figure 3B shows death rates according to VACS Index.

The cause of death was determined in 77.7% (21/27) of cases; the main causes were drug-related in 40% (*n* = 8; 5 overdoses, 3 unattended deaths in the context of current drug use), cancer in 19% (*n* = 4), infectious diseases in 9.5% (*n* = 2), cardiovascular diseases in 9.5% (*n* = 2), and liver cirrhosis in 9.5% (*n* = 2).

## Discussion

This longitudinal study in patients with CUD who were followed for 12 years confirms the prognostic value of a comorbidity index in predicting the risk of hospitalization and death in patients seeking treatment for the disorder. VACS is a multiorgan system injury index validated in 2013 for HIV-positive patients, although it has been described as a reliable index for HIV-negative patients as well and as a predictor of health outcomes such as hospitalization (Tate et al., 2013; Barakat et al., 2015; Hotton et al., 2017). To the best of our knowledge, this is the first time that VACS Index was analyzed in a cohort of HIV-positive and HIV-negative patients with CUD. Our results support the use of medical comorbidity rates in patients with SUD who start treatment, although more studies are required to confirm these findings.

In this study, VACS Index at baseline reflected moderate organ system damage, even though 47 and 25% of the patients had HCV and HIV infection, respectively. Despite moderate comorbidity, those with a VACS Index score over 40 were up to 2.6 times more likely to require hospitalization than those with a VACS score under 20. On the other hand, women with CUD and AUD were at a higher risk of hospitalization. Concomitant AUD is frequent in CUD and has been described as an indicator of poor health outcomes (Timko et al., 2018). Specifically, cocaethylene, the metabolite resulting from the concomitant use of alcohol and cocaine, has a known toxicity (Jones, 2019). This potent stimulant is more toxic than cocaine itself and has a longer half-life. On the other hand, the increased risk of hospitalization for women with CUD requires an accurate evaluation of the continuum of care and care coordination after discharge.

It was interesting to confirm that medical comorbidity was the only predictor of death in this cohort with a high prevalence of polysubstance use. Some studies on CUD indicate that the risk of death is higher in men, in those with a history of injected drugs, in those with an early onset of use, in those who drink alcohol, or in those with psychiatric comorbidity (Arendt et al., 2011; de la Fuente et al., 2014). However, there are hardly any studies on the medical comorbidity of CUD other than HIV infection and HCV infection. VACS Index analyzes kidney and liver function in addition to age, hemoglobin and HIV and HCV infections, thus reflecting the general health status. The recent 2019 version includes albumin, blood cell count, and BMI (Tate et al., 2019), which may improve the prognostic value of VACS.

It was interesting to note that BMI was below 19.5 kg/m^2^ in 50% of the patients in this study and below 16.4 kg/m^2^ in 25%, which indicates that being underweight might be associated with CUD or an underlying disease. A population study in adults aged 18–45 years demonstrated that the BMI of cocaine users is lower than that of non-users, although those BMI values were much higher than those observed in this study (Qureshi et al., 2014).

On the other hand, death rates in this hospital-based cohort were higher than that reported by another Spanish study of patients recruited in outpatient clinics (de la Fuente et al., 2014), although clearly lower than that reported by our group in past decades (Sanvisens et al., 2014).

ED admissions for accidents/injuries and non-specific symptoms were the most frequent during follow-up, suggesting that they could be related to continued substance use or complications derived from such use. The first systematic review and meta-analysis on healthcare utilization in patients with SUD was published in 2019 and shows that hospitalization and ED admissions are 5 and 7 times more frequent, respectively, in this group than in the general population (Lewer et al., 2020). In Spain, 38.4% of drug-related ED admissions can be attributed to cocaine (Miró et al., 2019). Furthermore, a study reveals that 18% of those admitted to an ED for cocaine use are readmitted in the following year (Miro et al., 2010).

Mental health-related complications, accidents/injuries, respiratory/lung related conditions, and digestive/liver diseases were other diagnoses frequently observed during follow-up. About 40% of the episodes were related to SUD either due to an associated mental illness or to trauma/accidents associated with substance use. These results are consistent with those presented in individuals who use illicit drugs (Aitken et al., 2013; Kendall et al., 2017). The pulmonary complications resulting from cocaine use could be due to several reasons, although the route of administration is relevant (Mégarbane and Chevillard, 2013); cocaine's respiratory toxicity can be immediate (i.e., acute lung injury or hypersensitivity reaction) or delayed (i.e., chronic obstructive pulmonary disease, cancer) (Mégarbane and Chevillard, 2013). A recent study shows that cocaine users are at increased risk for pulmonary hypertension (Alzghoul et al., 2020).

In terms of digestive/liver comorbidity, our findings are consistent with those observed in other studies (Pavarin et al., 2011). Liver decompensation was another frequent reason for clinical attention; however, a study in patients coinfected with HIV and HCV was unable to demonstrate an association between cocaine/crack use and evolution of liver fibrosis (Martel-Laferrière et al., 2017). Therefore, it is likely that alcohol abuse in the patients could explain those findings.

Cardiovascular complications in this long-term followed-up cohort were less frequent than expected, despite the extensive scientific literature on CUD and acute coronary syndrome (Lippi et al., 2010; Carrillo et al., 2011). However, the results are consistent with those reported in other cohorts with low frequency of coronary ischemic complications (Qureshi et al., 2014).

This study has several limitations that should be mentioned. First, socioeconomic and baseline psychiatric comorbidity data were not available, which could have facilitated the interpretation of some findings during follow-up. Second, temporary changes in cocaine use (i.e., remission or exacerbation of use) were not analyzed. In this sense, retention in care is critical for achieving remission of CUD. The high rate of ED admissions of the patients in this study and the diagnoses of episodes related to continued substance use suggest low treatment retention. Third, patients from this study were evaluated with different DSM versions; however, criteria for admission were similar throughout the study period and mainly related to the severity of CUD. Fourth, this study had a limited number of patients, which impairs the interpretation of some associations due to lack of statistical power (i.e., route of cocaine administration). In addition, this study was carried out in a single unit which limits the generalization of the findings.

In contrast, the strength of this study among patients seeking treatment for CUD highlights the challenges in measuring medical comorbidity with an index that has proven to be useful in the context of SUD. Most studies on cocaine-related morbidity are conducted in EDs with patients with acute intoxication (Arendt et al., 2011; Qureshi et al., 2014; Miró et al., 2019; Santurtún et al., 2020), which prevents an accurate clinical assessment of comorbidity. Finally, understanding the risk factors for mortality allows us to target preventive interventions to increase retention in care among those seeking treatment for the disorder.

## Data Availability

The datasets generated for this study are available on request to the corresponding author.
